# Changes in Cardiometabolic Risk Factors Following Nordic Walking in Adults with Prediabetes or Diabetes: A Systematic Review and Meta-Analysis of Pre–Post Interventions

**DOI:** 10.3390/life16071159

**Published:** 2026-07-13

**Authors:** Ankang Wu, Sichao Chen, Yubo Liu, Yong Zhang, Mallikarjuna Korivi, Weibing Ye

**Affiliations:** 1 College of Physical Education and Health Sciences, Zhejiang Normal University, Jinhua 321004, China; wuankang@zjnu.edu.cn (A.W.); csc0615@zjnu.edu.cn (S.C.); liuyubo0124@outlook.com (Y.L.); 2 Department of Rehabilitation Medicine, School of Medicine, Shaoxing University, Shaoxing 321000, China; 2021000025@usx.edu.cn

**Keywords:** pole walking, prediabetes, HbA1c, hyperglycemia, blood pressure

## Abstract

**Background:** This systematic review and meta-analysis examined the effect of Nordic walking (NW) on clinical outcomes, including anthropometrics, glycemic control, lipid profile, and blood pressure in adults with diabetes or prediabetes by comparing the changes within trials. **Methods:** PubMed, Web of Science, the Cochrane Library, Scopus, and Embase were searched. Studies reporting baseline and post-intervention data for NW in adults with diabetes or prediabetes were included. Meta-analysis was performed to calculate mean differences (MDs) with 95% confidence intervals (CIs). **Results:** Ten trials comprising 277 participants with diabetes or prediabetes were included. Meta-analysis showed that NW was associated with a significant reduction in body mass index (MD: −0.95 kg/m^2^, 95% CI: −1.65 to −0.25, *p* = 0.008) and waist circumference (MD: −2.23 cm, 95% CI: −4.04 to −0.42, *p* = 0.02), without changing bodyweight. Glycemic control favorably improved after NW, as represented by decreased HbA1c (MD: −0.24%, 95% CI: −0.45 to −0.04, *p* = 0.02), fasting blood glucose (MD: −10.65 mg/dL, 95% CI: −20.63 to −0.67, *p* = 0.04), and HOMA-IR (MD: −0.25, 95% CI: −0.47 to −0.04, *p* = 0.02). Furthermore, a significant decrease in both systolic blood pressure (MD: −6.44 mmHg, 95% CI: −10.15 to −2.73, *p* = 0.0007) and diastolic blood pressure (MD: −2.16 mmHg, 95% CI: −4.07 to −0.26, *p* = 0.03) was observed within the trial. However, none of the lipid profiles were affected by NW. **Conclusions:** As a non-pharmacological intervention, NW could be beneficial in reducing anthropometrics and blood pressure, while improving glycemic control in adults with diabetes or prediabetes.

## 1. Introduction

Nordic walking (NW) is a form of physical activity in which specifically designed poles are used to engage both upper and lower body muscle groups, and thereby reach the desired speed of walking [1,2]. The use of poles not only increases exercise intensity but also promotes energy expenditure [3,4]. These features make NW a popular and accessible alternative to traditional walking, particularly for the elderly with insufficient physical activity, arthritis, or other musculoskeletal disorders [5,6]. Several studies reported that NW is beneficial in improving health-related outcomes. For example, NW improves cognitive function in patients with Alzheimer’s disease [7], reduces depression and sleep disturbances in the elderly with depression disorders [8], and promotes cardiovascular performance and functional ability in patients with coronary artery disease [9]. NW also promotes glycemic control in people with overweight and obesity [10] and diabetes [11].

Type 2 diabetes (T2D) is a metabolic disorder characterized by elevated fasting blood glucose levels resulting from genetic, environmental, and/or lifestyle factors [12,13]. Prediabetes represents an intermediary stage in which fasting glucose levels are higher than the normal range (6.1–7.0 mmol/L), but below the diagnostic threshold for diabetes, and it typically progresses to T2D if not treated [14]. Currently, there are 589 million adults (20–79 years) with diabetes and 864.5 million adults with prediabetes worldwide [15]. Diabetes is associated with several complications, such as cardiovascular diseases (CVDs), nephropathy, retinopathy, and neuropathy, which ultimately increase the financial burden and risk of premature death [16,17]. Similar to diabetes, prediabetes or impaired glucose tolerance (IGT) is associated with obesity, dyslipidemia (higher triglycerides (TG) and/or lower high-density lipoprotein (HDL) cholesterol), and elevated blood pressure [18]. Given the high risk that adults with prediabetes are most likely to develop T2D in their lifetime, monitoring and controlling glycemic levels even in early phases is essential [19]. Non-pharmacological interventions, such as adopting healthy lifestyle behaviors, including regular physical activity, have been reported to be effective in treating or managing disease progression [20,21].

Previous studies have investigated the effect of NW on diabetes; however, the findings, particularly on glycemic control, a fundamental aspect in diabetes management, remain inconclusive [22]. Some randomized controlled trials (RCTs) have demonstrated that NW improves glycosylated hemoglobin (HbA1c) in adults with T2D [23,24], whereas others have shown no significant improvement [11,25]. Yet no prior systematic review and meta-analysis has specifically pooled within-group pre–post changes with NW in adults with prediabetes and diabetes. Although few meta-analyses evaluated the effect of NW in the elderly or adults with overweight/obesity, their findings on glycemic control or cardiovascular risk factors are conflicting. For instance, one meta-analysis of eight RCTs (*n* = 465) compared NW post-intervention changes with baseline, and reported a significant reduction in fasting blood glucose, homeostasis model assessment of insulin resistance (HOMA-IR), total cholesterol (TC), and TG in adults with overweight or obesity [26]. In contrast, another meta-analysis of 22 RCTs (*n* = 1271) reported that NW did not affect HbA1c in older adults, despite reducing bodyweight, body mass index (BMI), waist circumference, and fat percentage, compared to control [27]. These contradictory findings may be attributable to variations in intervention protocols, participant characteristics, and/or study designs in the included studies [28,29]. Heterogeneous populations with different analytical frameworks in these meta-analyses [26,27] limiting to draw definitive conclusions on clinical outcomes, specific to adults with prediabetes or diabetes.

These inconsistent findings from research studies [11,23,24,25] and meta-analyses [26,27] have hindered the integration of NW into exercise guidelines for adults with prediabetes or diabetes. Notably, no systematic review or meta-analysis has comprehensively evaluated the effect of NW on both glycemic control and cardiometabolic risk factors in this population. Therefore, this systematic review and meta-analysis was designed to synthesize the available evidence and clarify the extent to which NW is associated with favorable changes in cardiometabolic outcomes in adults with prediabetes or diabetes. Specifically, we pooled within-group changes in anthropometric measures, glycemic control, lipid profile, and blood pressure after NW intervention compared with pre-intervention values.

## 2. Materials and Methods

### 2.1. Search Strategy

We systematically searched PubMed, Scopus, Cochrane, EMBASE, and Web of Science databases from inception to December 2025. A comprehensive search strategy was performed using the combination of keywords related to intervention and population: “Nordic walking” OR” pole walking” OR “walking poles” “Nordic exercise” AND “type 2 diabetes” OR “diabetes mellitus, type 2” OR “diabetes mellitus” OR “T2D” OR “prediabetes” OR “impaired glucose tolerance” OR “insulin resistance” OR “HOMA-IR” OR “homeostasis model assessment of insulin resistance”. The search was conducted using one keyword from the intervention and another keyword from the population model, and the entire strategy was repeated by changing one keyword one time. No restriction on study design was applied during the search. The article search and review were conducted in accordance with the Preferred Reporting Items for Systematic Reviews and Meta-Analyses (PRISMA) 2020 guidelines [30,31]. The detailed search strategy for each database is provided as a Supplementary Table S1.

### 2.2. Eligibility Criteria

Studies were included if they met the following inclusion criteria: (1) participants should be diagnosed with type 2 diabetes or prediabetes; (2) participants must be adults aged 18 or above; (3) the intervention should be Nordic walking alone, and outcomes measured at baseline (pre) and after intervention (post); (4) the study should report at least one diabetes-related marker or cardiometabolic risk factors; (5) the intervention duration should be 4 weeks or above; and (6) studies published in English. Adults with prediabetes and T2D are both eligible for inclusion, as these two conditions are part of the same disease spectrum characterized by common pathophysiological features, including impaired glucose homeostasis, insulin resistance, and progressive β-cell dysfunction [32]. We excluded the studies based on the following criteria: (1) participants diagnosed with type 1 diabetes; (2) Nordic walking combined with other interventions; (3) lack of relevant outcomes or baseline data; and (4) studies with incomplete data. The search results were imported into EndNote 21, and duplicates were removed. Two review authors (A.W. and S.C.) independently reviewed the titles and abstracts of the articles, and any disputes were resolved by discussing with the corresponding authors (M.K. and W.Y.).

### 2.3. Data Extraction

Following careful screening and evaluation of the eligible full-text articles, two review authors (A.W. and S.C.) independently extracted and tabulated data. Uncertainties during data extraction were resolved by discussing with the corresponding authors (M.K. and W.Y.). The remaining authors (Y.L. and Y.Z.) helped in assessing, formatting, and validating the extracted data. The data were systematically collected using Excel spreadsheets that include study characteristics (first author, year of publication, study location), participants’ characteristics (sample size, mean age, sex, health status), intervention details (frequency, duration), and outcome measures (anthropometrics, diabetes biomarkers, lipid profiles, blood pressure, and VO_2max_). The complete details of the extracted data are presented in Table 1.

### 2.4. Quality Assessment

The quality of the included RCTs was assessed using the Cochrane Risk of Bias 2.0 (RoB2) tool [38]. The RoB2 tool evaluates the risk of bias across five domains, including (1) bias arising from the randomization process; (2) bias due to deviations from intended interventions. (3) bias due to missing outcome data; (4) bias in outcome measurement; and (5) bias in the selection of the reported results. Each included study was assessed for the five domains and labeled from D1 to D5, with the risk level represented by green (+), yellow (!), and red (−). Green indicates a low risk of bias, yellow indicates some concerns, and red indicates a high risk of bias.

The methodological quality of the non-RCTs was evaluated using the MINORS (Methodological Index for Non-Randomized Studies) scale, which employs a scoring system. The MINORS scale includes 12 evaluation items, each scored from 0 to 2, where 0 indicates no reporting, 1 indicates reporting with incomplete information, and 2 indicates reporting with sufficient information. Since the included studies lacked control trials, the first 8 items, which apply to studies without control groups, were assessed with a maximum total score of 16 points [39]. The quality of studies was assessed by two review authors (A.W. and S.C.), and any disagreements were resolved through discussion with the corresponding authors (M.K. and W.Y.). Publication bias was not formally assessed using Egger’s test, as the number of included studies in our analysis was insufficient (<10).

### 2.5. Statistical Analysis

A meta-analysis was conducted when three or more studies reported data on the same outcome. The pooled effect estimates were calculated using Review Manager (RevMan) version 5.4 (the Cochrane Collaboration, London, UK). The analysis was designed as a pre–post synthesis to estimate within-group changes, not as a comparative effectiveness analysis against a control trial. We included both RCTs and non-RCTs in the analysis and observed heterogeneous control comparators across the studies. These control conditions varied considerably, including structured exercise programs, habitual physical activity, physical activity counseling, gym-based exercise, or usual care (Supplementary Table S2). Given this substantial variability in control conditions, a comparative intervention versus control analysis was not methodologically robust and therefore was not undertaken. Instead, we performed a pre–post meta-analysis to synthesize within-group changes following NW. Due to the limited number of included studies, subgroup analysis based on disease type (prediabetes versus diabetes) was not performed.

The values for reported outcomes in NW trials (pre- and post-) were expressed as mean differences (MDs) with standard deviations (SDs), 95% confidence intervals (CIs), and the relevant forest plots were generated accordingly. For each outcome, the mean and standard deviation (SD) were extracted from each study. If the SD was not available for any outcome measure, it was calculated based on the standard error or CI of the group mean according to the methods described in the Cochrane Handbook for Systematic Reviews of Interventions. Heterogeneity for each outcome was assessed using the I^2^ statistic. The random-effects model was used if the I^2^ exceeds 50%, indicating substantial heterogeneity in that outcome. Alternatively, the fixed-effects model was used when the I^2^ is <50%, indicating low to moderate heterogeneity, as recommended by standard methodological guidance [40,41]. Statistical significance was set when *p* values are less than 0.05. Meta-analysis results of all outcomes are presented as forest plots where applicable.

## 3. Results

### 3.1. Selection of Studies

**Figure 1:**
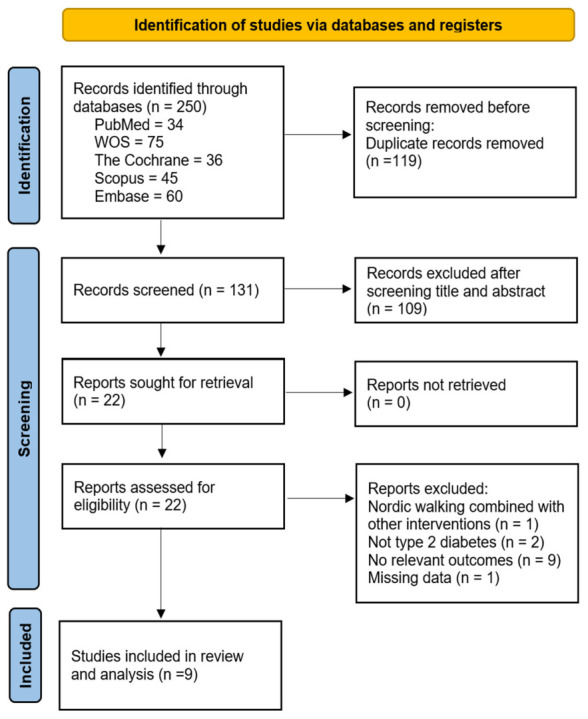
PRISMA flow diagram depicting the literature search, screening, and inclusion.

Through a systematic search across five major databases, we identified 250 records: PubMed (*n* = 34), Web of Science (*n* = 75), the Cochrane Library (*n* = 36), Scopus (*n* = 45), and Embase (*n* = 60). After the initial assessment, 119 duplicates were removed, and the remaining 131 articles were screened based on their titles and abstracts. Of these, 109 articles were excluded, as they did not meet our inclusion criteria. The full text of the remaining 22 articles was thoroughly assessed, and 13 were excluded for the following reasons: Nordic walking was combined with another intervention [42], participants were not T2D in two studies [43,44], lack of relevant outcome measures data in nine studies [45,46,47,48,49,50,51,52,53], and missing outcome data in one study [54]. Finally, nine articles reporting a total of 10 trials met our inclusion criteria and were included in the meta-analysis [11,23,24,25,33,34,35,36,37]. The study selection process and number of articles in each step are clearly indicated in the PRISMA flowchart (Figure 1).

### 3.2. Characteristics of Included Studies and Participants

The nine included articles, reporting 10 trials, comprised a total of 277 participants with type 2 diabetes or prediabetes. The sample consisted of 171 females (62% and 106 males (38%), with a mean age of 61.39 years. Six studies (seven trials) enrolled both male and female participants [11,24,25,33,36,37], two studies enrolled females only [23,35], and one study included males only [34]. Among the 10 trials, eight investigated the effect of NW on adults with diabetes (*n* = 224), and two investigated the effect on adults with IGT (*n* = 53). The duration of NW intervention is one month in one study [24], three months in five studies [23,34,35,36,37], and four months in two studies (3 trials) [11,33]. The NW session ranged from 45 to 90 min with a frequency of one to three times per week. Full details of the trials, intervention, and baseline characteristics of participants are listed in Table 1.

### 3.3. Risk of Bias Assessments

**Figure 2:**
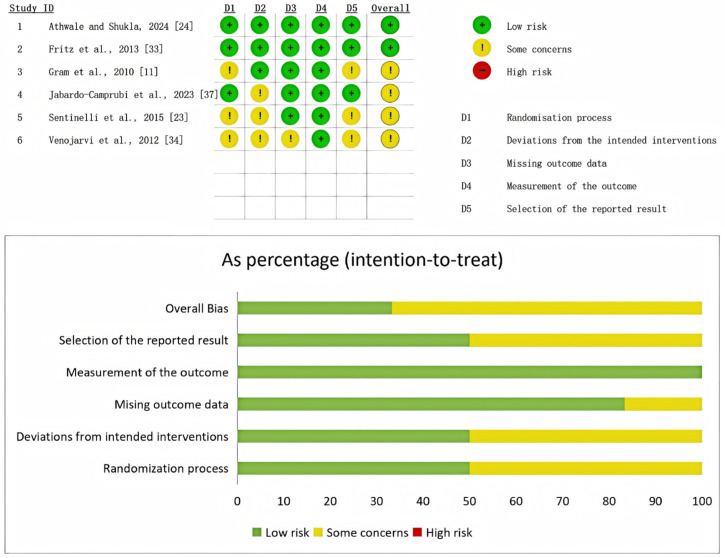
Risk of bias assessment for RCTs using the Cochrane RoB 2 tool.

The Cochrane RoB2 was used to assess the risk of bias in RCTs [38], while the MINORS was used for the non-RCTs [39]. Representative results are shown in Figure 2 and Table 2.

Among the six RCTs, two used computer-generated randomization sequences [24,37]. Although other studies reported the use of randomization, they have not provided sufficient methodological details, which may introduce the risk of selection bias. Three studies are considered to have a low risk of bias for deviations from intended intervention [11,24,33], and one study is identified with missing outcome data [34]. Regarding outcome measurement, all studies are considered to have a low risk of bias. Three studies showed some concerns for the selection of reported results [11,23,34]. Overall, two studies were assessed as having a low risk of bias, while four studies were considered to have some concerns for risk of bias.

Three studies were compared with non-randomized controlled trials, and their overall scores were 10, 13, and 10, respectively, out of a maximum possible score of 16 [25,35,36]. These studies clearly defined their research objectives, and each received a score of 1 for the item “inclusion of consecutive participants.” For “prospective data collection” and “clear evaluation of primary outcome criteria,” all three studies scored 2 points. Regarding the “objective evaluation of research outcomes,” one study [25] reported, but did not clarify, while the other study [35] did not report it. In contrast, Pippi et al. [36] provided a comprehensive and rigorous evaluation of the outcomes in this domain. For the “follow-up period consistent with research objectives,” two studies reported it [25,36], while the other had incomplete information [35]. For ‘dropout rate’, two studies had a rate below 5% [35,36], while the other had unclear information [25]. None of the studies reported prospective calculations of sample size. Overall, these three studies may have a certain risk of bias.

### 3.4. Nordic Walking Associated with Decreased BMI and Waist Circumference, but Not Bodyweight

This meta-analysis was designed and performed as a pre–post synthesis to estimate within-group changes from baseline to follow-up NW trial, rather than to estimate NW effect relative to a control. Therefore, the results presented in our study reflect within-trial changes and should be interpreted as descriptive associations rather than causal effects of NW intervention.

**Figure 3:**
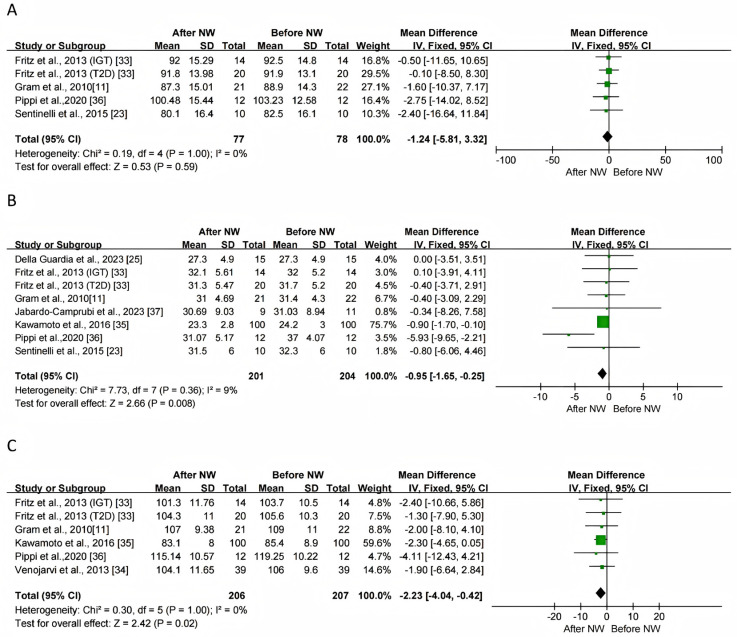
Forest comparing changes in anthropometric measures before and after Nordic walking in adults with prediabetes or diabetes. (**A**) bodyweight; (**B**) body mass index; and (**C**) waist circumference. Abbreviations: SD, standard deviation; CI, confidence interval.

Four studies (five trials) reported the effect of NW on bodyweight in adults with prediabetes or diabetes [11,23,33,36]. Pooled analysis showed that NW did not significantly decrease bodyweight compared to the pre-intervention values (Figure 3A, MD: −1.24 kg, 95% CI: −5.81 to 3.32, *p* = 0.59, *I*^2^ = 0%). The BMI data were reported in eight trials, and the analysis showed that NW significantly decreased BMI in adults with prediabetes or diabetes (Figure 3B, MD: −0.95 kg/m^2^, 95% CI: −1.65 to −0.25, *p* = 0.008, *I*^2^ = 9%). Similarly, waist circumference was significantly decreased after NW compared to their baseline values (MD: −2.23 cm, 95% CI: −4.04 to −0.42, *p* = 0.02), with *I*^2^ = 0% (Figure 3C). These findings indicate that NW is associated with favorable changes in BMI and waist circumference, but not in body weight, in adults with diabetes or prediabetes.

### 3.5. Changes in Diabetes Markers Following Nordic Walking

**Figure 4:**
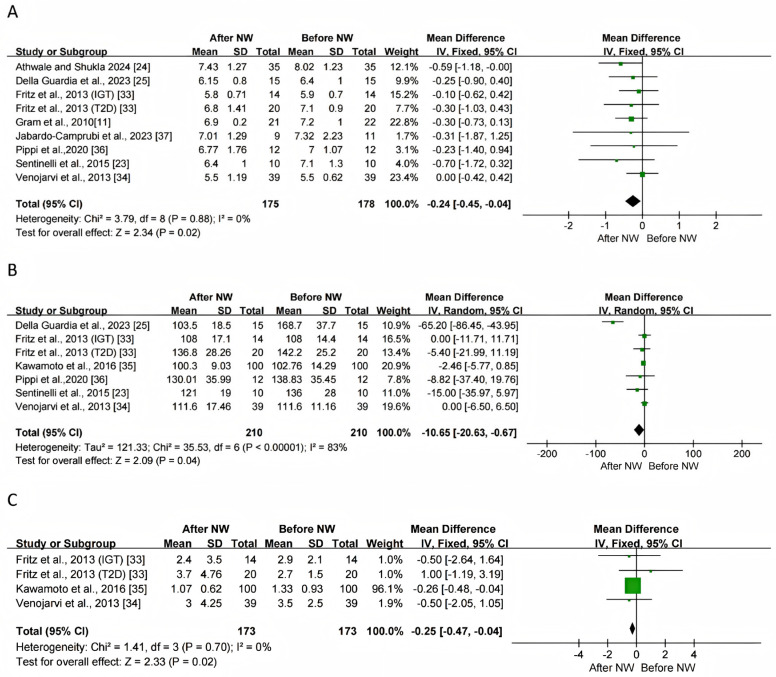
Forest plots comparing the differences in glycemic control markers before and after the Nordic walking intervention in adults with prediabetes or diabetes. (**A**) HbA1c; (**B**) fasting blood glucose; and (**C**) HOMA-IR. Abbreviations: SD, standard deviation; CI, confidence interval.

Nine trials from eight studies evaluated the effect of NW on HbA1c in adults with prediabetes or diabetes. The pooled analysis showed a significant reduction in HbA1c with NW compared with the baseline (Figure 4A, MD: −0.24%, 95% CI: −0.45 to −0.04, *p* = 0.02, *I*^2^ = 0%), indicating improvements following NW. Similarly, fasting blood glucose (7 trials) was also associated with a considerable decrease after NW (MD: −10.65 mg/dL, 95% CI: −20.63 to −0.67, *p* = 0.04). However, the heterogeneity for fasting blood glucose was higher (*I*^2^ = 83%, Figure 4B). We then performed a sensitivity analysis by sequentially excluding individual studies, and heterogeneity was eliminated (*I*^2^ = 0) upon excluding the study by Della Guardia et al. [25], in which the intervention duration differed from that of other included studies.

Furthermore, the HOMA-IR values (4 trials) were also reported to decrease after NW compared with pre-intervention values (Figure 4C, MD: −0.25, 95% CI: −0.47 to −0.04, *p* = 0.02, *I*^2^ = 0%). Taken together, the NW intervention is associated with lowering fasting blood glucose, HOMA-IR, and HbA1c levels in adults with prediabetes or diabetes.

### 3.6. Effect of Nordic Walking on Lipid Profiles

**Figure 5:**
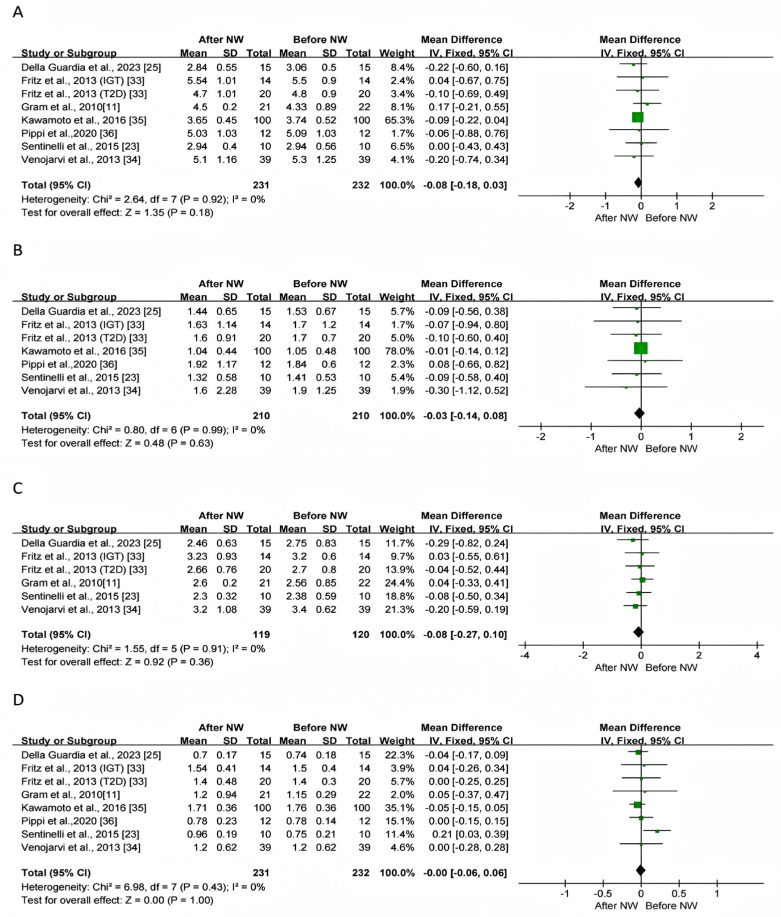
Forest plots comparing the changes in lipid profiles before and after Nordic walking in adults with prediabetes or diabetes. (**A**) total cholesterol; (**B**) triglycerides; (**C**) low-density lipoprotein cholesterol; and (**D**) high-density lipoprotein cholesterol. Abbreviations: SD, standard deviation; CI, confidence interval.

The effect of NW on CVD risk factors, including TC, TG, low-density lipoprotein (LDL) cholesterol, and HDL cholesterol, was assessed by meta-analysis. Changes in TC are reported across eight trials (*n* = 463). Pooled results showed that NW did not significantly alter TC levels in participants with prediabetes or diabetes when compared to baseline values (Figure 5A, MD: −0.08, 95% CI: −0.18 to 0.03, *p* = 0.18, *I*^2^ = 0%). Similarly, the changes in TG, reported in seven trials, were not significant after NW (Figure 5B, MD: −0.03, 95% CI: −0.14 to 0.08, *p* = 0.63, *I*^2^ = 0%). Next, LDL cholesterol (6 trials) and HDL cholesterol (8 trials) levels were also not significantly changed after NW (LDL: MD: −0.08, 95% CI: −0.27 to 0.10, *p* = 0.36, *I*^2^ = 0%, Figure 5C. HDL: MD: −0.00, 95% CI: −0.06 to 0.06, *p* = 1.00, *I*^2^ = 0%. Figure 5D). Overall, these findings suggest that NW at the given duration (1 to 6 months) appears not to be associated with improved lipid profiles in adults with prediabetes or diabetes.

### 3.7. Nordic Walking Associated with Improved Blood Pressure

**Figure 6:**
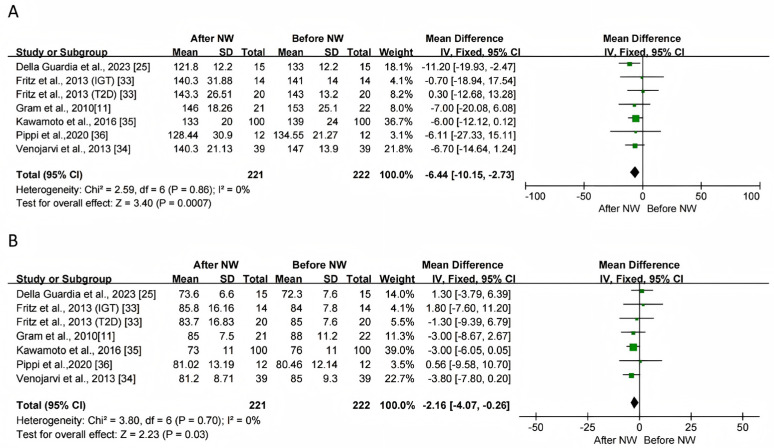
Forest plots comparing the changes in blood pressure before and after Nordic walking intervention in adults with prediabetes or diabetes. (**A**) systolic blood pressure; (**B**) diastolic blood pressure. Abbreviations: SD, standard deviation; CI, confidence interval.

A meta-analysis was conducted to evaluate within-group changes in systolic blood pressure (SBP) and diastolic blood pressure (DBP) following NW in adults with prediabetes or diabetes. Seven trials reported the changes in SBP and DBP with NW, and the changes were compared with pre-intervention values. The pooled analysis revealed that NW was associated with a significant reduction in SBP in adults with prediabetes or diabetes (Figure 6A, MD: −6.44 mmHg, 95% CI: −10.15 to −2.73, *p* = 0.0007, *I*^2^ = 0%). The observed SBP reduction (−6.44 mmHg) suggests the clinical significance of NW, and such a reduction is associated with decreased risk of CVDs [55,56]. Similarly, a significant decrease in DBP was also observed after NW (Figure 6B, MD: −2.16 mmHg, 95% CI: −4.07 to −0.26, *p* = 0.03, *I*^2^ = 0%). These results indicate that NW intervention is associated with favorable changes in both SBP and DBP in adults with prediabetes or diabetes.

### 3.8. Sensitivity Analysis

We observed high heterogeneity of fasting blood glucose data (I^2^ = 83%, *p* < 0.00001). To identify the potential source of heterogeneity, subgroup analysis or meta-regression are regarded as effective approaches [57]. However, given the limited number of included studies, we were unable to conduct these analyses. Instead, sensitivity analysis (leave-one-out) was conducted to evaluate the robustness of the pooled results and to identify potential sources of heterogeneity. We observed that the heterogeneity was eliminated (I^2^ = 0%) upon excluding the study by Della Guardia and colleagues [25]. This study differed from other included studies in terms of intervention duration (6 months), which may have contributed to the observed heterogeneity. Sensitivity analyses using a consistent random-effects model for all outcomes yielded results that were largely consistent in both effect direction and statistical significance with the primary analyses, indicating that the pooled findings are robust to the choice of statistical model.

## 4. Discussion

Systematic reviews and meta-analyses are fundamental to the evidence-based evaluation of interventions, as they establish the strength of evidence and quantify the magnitude of treatment effects [58]. Existing syntheses on the effects of NW are equivocal, likely due to disparities in participants’ health status or intervention protocols. Critically, none of the analyses specifically focused on adults with prediabetes or diabetes. To the best of our knowledge, this is the first systematic review and meta-analysis to compare the pre- and post-intervention effects of NW on glycemic control and cardiometabolic risk factors in this population. Our analyses demonstrated that NW is associated with a reduction in BMI and waist circumference compared with baseline. This improvement in anthropometrics appears to be accompanied by notable reductions in HbA1c, fasting blood glucose, and HOMA-IR, which emphasizes the beneficial effects of NW. Furthermore, NW was associated with reductions in both SBP and DBP, while lipid profile remained unchanged. Taken together, these findings suggest that NW is associated with improved anthropometrics, glycemic control, and blood pressure in adults with prediabetes or diabetes.

Anthropometric measures, such as body weight, BMI, or waist circumference, are key clinical variables for assessing health status and predicting the risk of metabolic disorders and diabetes [59,60]. In our analysis, NW was associated with a considerable decrease in BMI and waist circumference in adults with prediabetes or diabetes, without a significant change in body weight. Regarding the clinical relevance of observed BMI reduction, recent guidelines suggested that a reduction of 1.0 kg/m^2^ may represent a clinically meaningful threshold [61]. Clinically, any reduction in BMI is said to be associated with reduced risk of microvascular and macrovascular complications in adults with obesity and diabetes [62]. Although a 0.95 kg/m^2^ BMI reduction was observed in our study, this could not reach a clinically significant threshold. Fritz et al. reported that 4-month NW significantly decreased BMI in adults with normal glucose tolerance, but not in those with prediabetes or diabetes [45]. Another study reported that reduced waist circumference after NW is accompanied by a reduced body adiposity index in women with metabolic disorders [43]. Given that higher BMI and waist circumference values are strongly correlated with the incidence or progression of diabetes [63,64], reductions in these variables indicate the beneficial effect of NW in preventing diabetes progression. The efficacy of NW in improving BMI and waist circumference may be attributed to its unique biomechanics or energy expenditure during intervention. The use of specialized poles engages upper-body musculature in addition to the legs, activating a greater muscle mass compared with conventional walking. This increased muscle engagement leads to increased energy expenditure, which promotes mobilization of fat reserves, particularly visceral and abdominal fat [23,27,65]. A recent meta-analysis concluded that NW can decrease several CVD risk factors, including body weight, BMI, and waist circumference in older adults; however, the included population in this analysis was heterogeneous with different health statuses [27]. Although NW decreased both BMI and waist circumference, the absence of a significant change in body weight is an interesting finding that warrants future investigations.

One of the key findings of our study is that NW is associated with improved glycemic control in adults with prediabetes or diabetes, evidenced by a significant decrease in fasting blood glucose, HbA1c, and HOMA-IR compared with baseline. Elevated blood glucose concentration is a primary risk factor for prediabetes or CVD, whereas decreased blood glucose with NW or aerobic exercise is associated with improved whole-body insulin sensitivity, delayed onset of diabetes, and reduced CVD risk [20,66,67]. However, substantial heterogeneity observed for fasting blood glucose (I^2^ = 83%) implies careful consideration. Due to a lack of the required number of studies (10), we were unable to perform a formal meta-regression or subgroup analysis to identify the source of heterogeneity [57]. We assume that the high heterogeneity may be due to the variance in basic characteristics of participants across studies, like prediabetes versus diabetes status. Variations in intervention protocols, frequency, intensity, and duration of NW may have influenced the magnitude of glycemic changes. In addition, differences in study designs, including single-arm versus RCTs and adherence to the protocol, may have led to considerable heterogeneity. The sensitivity analysis suggests that one study with variations in intervention duration (6 months) may be a potential factor for heterogeneity [25].

HbA1c reflects the mean blood glucose concentrations over 2 to 3 months and is strongly correlated with the incidence or progression of diabetes and its complications. Therefore, measuring HbA1c is considered the gold standard for monitoring chronic hyperglycemia in patients with diabetes or prediabetes, and HbA1c values are clinically validated to determine the effectiveness of interventions [68,69,70]. Given that maintaining lower HbA1c levels (<7%) is clinically significant [71,72], the reductions in HbA1c with NW, particularly in this population, underscore its importance in mitigating hyperglycemia-associated complications. The efficacy of NW in lowering HbA1c aligns with established evidence on aerobic exercise and may be attributed to sustained improvements in metabolic function and enhanced glucose disposal [73,74]. In contrast, NW duration did not improve HbA1c in older adults with T2D, despite significantly decreased blood glucose levels [25]. However, NW-induced improvement in short-term and long-term glucose levels in our study is accompanied by improved insulin resistance (HOMA-IR). The beneficial effect of NW on glycemic control is consistent with other meta-analyses, which show that different exercise modalities, including regular walking or resistance training, have been demonstrated to improve glycemic control in adults with diabetes or metabolic disorders [75,76,77]. The underlying physiological mechanism may involve enhanced skeletal muscle glucose uptake through contraction-stimulated GLUT4 translocation and activation of AMP-activated protein kinase (AMPK) related signaling pathways [78,79]. Importantly, exercise-stimulated glucose uptake remains largely preserved despite impaired insulin signaling in individuals with T2D, suggesting that NW may improve glycemic control through insulin-independent pathways that bypass defects in insulin action [80]. Taken together, our findings suggest that NW represents a beneficial non-pharmacological approach for improving glycemic control in individuals at risk of diabetes.

Although we observed a statistically significant reduction in HbA1c (−0.24%) after NW, this falls below the commonly considered threshold for the minimal clinically important difference (MCID) [81,82]. A systematic review and meta-analysis reported that a pooled reduction of −0.31% in HbA1c in diabetes met the threshold of 0.3% proposed by the European Medicines Agency as clinically relevant for risk reduction in diabetic complications [83]. The observed −0.24% HbA1c reduction in our study does not consistently reach the MCID threshold, but it is worth noting that such a modest reduction may contribute to mitigating chronic diabetes complications. The UK Prospective Diabetes Study demonstrated that every 1% reduction in HbA1c is associated with a 21% reduction in diabetes-related deaths and a 37% reduction in microvascular complications [84]. Therefore, even the modest magnitude of HbA1c reduction in our study may be considered clinically relevant when sustained over time, particularly for adults with prediabetes or early-stage T2D. Another meta-analysis used an MCID of 0.05 for HOMA-IR and reported that pooled mean differences of −0.14 were considered “clinically important benefits” [85]. We found a reduction of −0.25 in HOMA-IR in our analysis, which is larger than the MCID threshold, suggesting improvement in insulin sensitivity achieved through NW is not only statistically significant but also clinically meaningful.

Despite the beneficial effects of NW on anthropometrics and glycemic control, it appears that NW is not associated with improved lipid profiles, TC, TG, LDL, or HDL cholesterol, indicating that NW alone had no influence on lipid profile in this population. These findings aligned with a study conducted on overweight adults with normal glucose tolerance, prediabetes, or diabetes, which reported that NW (4 months) did not change TC, TG, LDL, or HDL levels within the prediabetes or diabetes groups [33]. Similarly, a 12-week NW intervention in older women with several CVD risk factors showed no effect in TG and LDL cholesterol, despite decreased TC and HDL cholesterol [35]. Even a longer duration of NW (6 months) was also reported to be ineffective in improving TC, TG, HDL, and LDL cholesterol in older patients with diabetes [25]. In contrast, Prusik et al. demonstrated improved lipid profiles when NW was combined with vitamin D in elderly women [86], while NW combined with time-restricted eating did not affect lipid profiles in women with overweight and obesity [87].

These inconsistent results of NW on lipid profiles were further extended to meta-analyses. For instance, a meta-analysis on the elderly population claimed beneficial effects of NW on improving cardiovascular outcomes and lipid profile; however, the number of included RCTs and the provided statistical evidence for the individual outcomes were inadequate [88]. Another recent meta-analysis of 22 RCTs showed that NW significantly decreased TC, TG, and LDL cholesterol, while HDL cholesterol remained unchanged in older adults, irrespective of their health status [27]. Such discrepancies are likely attributable to substantial variations in study designs, participants’ characteristics, and/or NW protocol. The observed differences, particularly in glycemic and lipid profile outcomes, may arise from the distinct physiological responsiveness of glucose and lipid metabolism to exercise intervention. Exercise-induced favorable changes in lipid metabolism may require a minimum intensity threshold to effectively mobilize and oxidize fatty acids [89,90]. The intensity of NW in our included studies was unclear. We assume that the intensity of NW may have been below the individual’s maximum rate of fat oxidation (Fatmax), resulting in insufficient lipid mobilization to induce meaningful changes in circulating lipid levels [89,90]. Previous studies have indicated that training at intensities corresponding to the dominance of lipid metabolism is necessary to achieve a significant reduction in TG and TC levels [90,91]. TGs stored in adipose tissue are rapidly mobilized for energy metabolism when glucose is insufficient as a fuel source during exercise; however, significant TG reduction may require regular and continuous high-intensity exercise [92]. Greater stimuli from high-intensity exercise may be required for a significant reduction in TG and LDL levels [93,94]. Additionally, given the established relationship between body fat and cholesterol, a greater decrease in fat percentage with exercise may favorably improve lipid profiles [95]. In light of these null effects of NW, further RCTs in individuals with prediabetes or diabetes, as well as subsequent systematic reviews and meta-analyses, are warranted.

Effective management of blood pressure is crucial in individuals with prediabetes or diabetes, given that elevated blood pressure or hypertension is a primary driver of CVDs and other metabolic complications [96,97,98]. A notable finding in our meta-analysis is that NW is associated with a substantial reduction in both SBP and DBP in adults with prediabetes or diabetes. The observed reduction of –6.44 mmHg in SBP is clinically relevant, as a 5 mmHg reduction in SBP has been associated with a 14% reduction in stroke risk and a 9% reduction in coronary heart disease risk at a population level [55,99]. Furthermore, it has been highlighted that a 2 mmHg lower SBP is associated with approximately 10% lower stroke mortality and about 7% lower mortality in middle-aged adults with vascular complications [56]. Our findings align with existing evidence from meta-analysis, revealing that NW considerably decreased DBP and cardiovascular risk factors in older adults [27]. However, the evidence remains inconsistent across the studies. One RCT showed that NW did not affect blood pressure in adults with prediabetes or diabetes [33], and a systematic review and meta-analysis reported that neither SBP nor DBP improved with NW in adults with overweight and obesity [26]. Nevertheless, NW is said to be a suitable complementary strategy for improving SBP (but not DBP), along with enhanced physical and mental health in older patients with diabetes [25]. The antihypertensive effects of NW observed in our study are noteworthy, as the magnitude of SBP reduction is greater and comparable to other exercise modes reported in a previous meta-analysis [74]. The potential mechanisms underlying these blood pressure improvements with exercise may include enhanced endothelial function, nitric oxide bioavailability, reduced systemic vascular resistance, and improved autonomic regulation [100,101]. Taken together, our findings suggest that NW may represent a practical, non-pharmacological approach for adults with prediabetes or diabetes who are at risk of hypertension. However, given the nature of our analysis (pre–post), these findings should be interpreted as associations rather than causal effects, and further confirmatory studies are warranted.

In the context of the methodological quality of the included studies, the interpretation of our pooled estimates should be considered cautiously. The risk of bias assessment revealed that the ‘overall bias’ for most of the RCTs was classified as having ‘some concern’, while two studies [24,33] were judged to have low risk of bias across all domains. The methodological limitations, particularly the lack of adequate randomization details and potential selective reporting bias, possibly influenced the magnitude and direction of the observed effect estimates. Studies with unclear randomization methods or selective reporting are more susceptible to bias, potentially overestimating the intervention effects [102,103]. Therefore, despite the favorable associations of NW with clinical outcomes, the pooled estimates should be interpreted with caution, and the results are best considered hypothesis-generating rather than confirmatory.

### Limitations

Our meta-analysis included both RCTs and non-RCTs, which could introduce selection bias and affect the reliability of the findings. For instance, in non-RCTs, participants may not have been randomly assigned to the NW trial, potentially leading to differences in baseline characteristics between groups, which could affect the interpretation of outcomes. Due to substantial variability in control conditions, we were unable to conduct a comparative intervention versus control analysis. Instead, a pre–post meta-analysis was performed to synthesize within-group changes following NW, which limits our ability to attribute the results solely to NW. Most RCTs were classified as having ‘some concerns’ for risk of bias, which may affect the reliability of the pooled estimates. Some included studies had small sample sizes, which may result in insufficient statistical power to detect the true effects. For example, when assessing the effect of NW on lipid levels, limited sample sizes may hinder accurate evaluation of its potential benefits. Due to the lack of the required number of studies (>10), publication bias could not be assessed using Egger’s test. Additionally, statistical power could not be reliably calculated due to the limited number of studies; therefore, the findings should be interpreted with appropriate caution. Considerable heterogeneity was observed for fasting blood glucose, indicating variations in participants’ characteristics, intervention protocols, or study designs. Although sensitivity analysis identified one study as a potential source of heterogeneity, the small number of included studies precludes a formal subgroup analysis. Therefore, excluding individual studies alone may not fully explain the sources of heterogeneity.

Furthermore, because this meta-analysis was based on within-group pre–post comparisons, the observed improvements may have been partly influenced by time-related factors (lifestyle modifications, medication adjustments, or natural disease progression) and cannot be attributed entirely to NW. Information regarding the stability of the glucose-lowering medication regimen was not consistently reported across the included studies. The absence of this information may affect the interpretation of the observed changes in glycemic control. As prediabetes and diabetes differ in glycemic severity, the pooled estimates may not fully address the population-specific responses to NW. Finally, the generalizability of our findings is limited by the small number of studies and the heterogeneity in participant characteristics and intervention protocols. Future meta-analyses with a larger number of studies (especially RCTs) are required to conduct subgroup analysis to explore potential moderators of the NW (intervention duration, age, sex, or diabetes versus prediabetes).

## 5. Conclusions

In our systematic review and meta-analysis, we observed that Nordic walking intervention is associated with improved anthropometrics, glycemic control, and blood pressure compared with baseline in adults with prediabetes or diabetes. Our findings suggest the integration of NW into standard exercise regimens could be a beneficial approach to promote metabolic health in this population, who are at risk of developing CVDs. Further studies are encouraged to explore the underlying physiological adaptations responsible for these beneficial effects, which may assist in designing personalized NW intervention programs based on individuals’ needs and physical fitness levels.

## Figures and Tables

**Table 1:** Characteristics of the included studies.

Study Details		Participants’ Characteristics			Intervention Protocols		Outcomes
Author/Years	Country	Health Status (N)	Sex (m/f)	Age (Years)	Time/Frequency	Duration	
Gram et al., 2010 [11]	Denmark	T2D (22)	10/12	62 ± 10	45 min/session 2 times/week first 2 months 1 time/week next 2 months	4 months	Body weight, BMI, VO_2_max, WC, HbA1c, SBP, DBP, Hip circumference, TC, HDL, LDL
Fritz et al., 2013 (IGT) [33]	Sweden	IGT (14)	5/9	59.1 ± 6.2	5 h/week	4 months	Body weight, BMI, WC, SBP, DPB, p-glucose fasting, p-glucose 2h-post-load, HbA1c, HOMA-IR, TC, TG, HDL, LDL, Peak VO2.
Fritz et al., 2013 (T2D) [33]	Sweden	T2D (20)	13/7	61.4 ± 4.6	5 h/week	4 months	Body weight, BMI, Waist, SBP, DPB, p-glucose fasting, p-glucose 2h-post-load, HbA1c, HOMA-IR, TC, TG, HDL, LDL, Peak VO2.
Venojarvi et al., 2013 [34]	Finland	IGT (39)	39/0	55 ± 6.2	60 min/session 3 times/week	3 months	Body weight, FBG, 2 h glucose, fasting insulin, TC, TG, FLI, 2-h insulin, HbA1c, SBP, DBP
Sentinelli et al., 2015 [23]	Italy	T2D (10)	0/10	54 ± 9	60–90 min/session 3 times/week	3 months	Weight, BMI, HbA1c, HDL, TG, TC, FBG, handgrip test
Kawamoto et al., 2016 [35]	Japan	T2D (100)	0/100	68 ± 7	120 min/week	3 months	BMI, WC, SBP, DBP, TC, HDL, LDL, TG, FPG, HOMA-IR
Pippi et al., 2020 [36]	Italy	T2D (12)	6/6	58 ± 6.35	90 min/session 2 times/week	3 months	Body weight, WC, BMI, SBP, DBP, HbA1c, FBG, TC, TG, VO2max
Jabardo-Camprubi et al., 2023 [37]	Spain	T2D (10)	6/4	69.5 ± 4.9	2 times/week	3 months	WC, HbA1c, BMI
Della Guardia et al., 2023 [25]	Italy	T2D (15)	10/5	65 ± 6.2	60 min/session 2 times/week	6 months	BMI, FBG, HbA1c, SBP, DBP, HDL, LDL, TG, TC
Athwale and Shukla 2024 [24]	India	T2D (35)	17/18	49.57 ± 7.2	45 min/session 5 times/week	1 month	HbA1c, VO_2_max

N, number; m/f, male/female; T2D, type 2 diabetes; BMI, body mass index; WC, waist circumference; VO_2_max, maximum oxygen consumption; HbA1c, glycosylated hemoglobin; SBP, systolic blood pressure; DBP, diastolic blood pressure; TC, total cholesterol; TG, triglycerides; HDL, high-density lipoprotein; LDL, low-density lipoprotein; IGT, impaired glucose tolerance; HOMA-IR, Homeostatic model assessment of insulin resistance; FBG, fasting blood glucose.

**Table 2:** Quality assessment for non-RCTs using MINORS.

Evaluation	Study		
	Della Guardia et al., 2023 [25]	Kawamoto et al., 2016 [35]	Pippi et al., 2020 [36]
Clear objectives	2	2	2
Include consecutive patients	1	1	1
Prospective data collection	2	2	2
Endpoint suitable for research purposes	2	2	2
Fair evaluation of research endpoints	1	0	2
Follow-up period suitable for research purposes	2	1	2
The rate of lost visits is less than 5%	1	2	2
Prospective calculation of research scale	0	0	0
Total score	10	10	13

## Data Availability

The datasets generated and/or analyzed during the current study are available from the corresponding author on reasonable request.

## Supplementary Materials

The following supporting information can be downloaded at: https://www.mdpi.com/article/10.3390/life16071159/s1, Table S1: Detailed search strategy for each database; Table S2: Comparator conditions of the included studies.

## References

[1] Shim J.-m., Kwon H.-y., Kim H.-r., Kim B.-i., Jung J.-h. (2013). Comparison of the Effects of Walking with and Without Nordic Pole on Upper Extremity and Lower Extremity Muscle Activation. J. Phys. Ther. Sci..

[2] Schiffer T., Knicker A., Montanarella M., Strüder H.K. (2010). Mechanical and physiological effects of varying pole weights during Nordic walking compared to walking. Eur. J. Appl. Physiol..

[3] Pellegrini B., Peyré-Tartaruga L.A., Zoppirolli C., Bortolan L., Bacchi E., Figard-Fabre H., Schena F. (2015). Exploring Muscle Activation During Nordic Walking: A Comparison Between Conventional and Uphill Walking. PLoS ONE.

[4] Hansen E.A., Smith G. (2009). Energy expenditure and comfort during Nordic walking with different pole lengths. J. Strength Cond. Res..

[5] Parkatti T., Perttunen J., Wacker P. (2012). Improvements in functional capacity from Nordic walking: A randomized-controlled trial among elderly people. J. Aging Phys..

[6] Bieler T., Siersma V., Magnusson S.P., Kjaer M., Christensen H.E., Beyer N. (2016). In hip osteoarthritis, Nordic Walking is superior to strength training and home-based exercise for improving function. Scand. J. Med. Sci. Sports.

[7] Angiolillo A., Leccese D., Ciccotelli S., Di Cesare G., D’Elia K., Aurisano N., Matrone C., Dentizzi C., Di Costanzo A. (2023). Effects of Nordic walking in Alzheimer’s disease: A single-blind randomized controlled clinical trial. Heliyon.

[8] Park S.D., Yu S.H. (2015). The effects of Nordic and general walking on depression disorder patients’ depression, sleep, and body composition. J. Phys. Ther. Sci..

[9] Nagyova I., Jendrichovsky M., Kucinsky R., Lachytova M., Rus V. (2020). Effects of Nordic walking on cardiovascular performance and quality of life in coronary artery disease. Eur. J. Phys. Rehabil. Med..

[10] Hagner-Derengowska M., Kałużny K., Kochański B., Hagner W., Borkowska A., Czamara A., Budzyński J. (2015). Effects of Nordic Walking and Pilates exercise programs on blood glucose and lipid profile in overweight and obese postmenopausal women in an experimental, nonrandomized, open-label, prospective controlled trial. Menopause.

[11] Gram B., Christensen R., Christiansen C., Gram J. (2010). Effects of nordic walking and exercise in type 2 diabetes mellitus: A randomized controlled trial. Clin. J. Sport Med..

[12] Galicia-Garcia U., Benito-Vicente A., Jebari S., Larrea-Sebal A., Siddiqi H., Uribe K.B., Ostolaza H., Martín C. (2020). Pathophysiology of Type 2 Diabetes Mellitus. Int. J. Mol. Sci..

[13] Arslan A.K., Yagin F.H., Algarni A., Karaaslan E., Al-Hashem F., Ardigò L.P. (2024). Enhancing type 2 diabetes mellitus prediction by integrating metabolomics and tree-based boosting approaches. Front. Endocrinol..

[14] Diagnosis T.E.C.o.t., Mellitus C.o.D. (2003). Report of the Expert Committee on the Diagnosis and Classification of Diabetes Mellitus. Diabetes Care.

[15] International Diabetes Federation IDF Diabetes Atlas 11th Edition. https://idf.org/about-diabetes/diabetes-facts-figures/.

[16] Farmaki P., Damaskos C., Garmpis N., Garmpi A., Savvanis S., Diamantis E. (2020). Complications of the Type 2 Diabetes Mellitus. Curr. Cardiol. Rev..

[17] Scilletta S., Di Marco M., Miano N., Capuccio S., Musmeci M., Bosco G., Di Giacomo Barbagallo F., Martedì M., La Rocca F., Vitale A. (2025). Cardiovascular risk profile in subjects with diabetes: Is SCORE2-Diabetes reliable?. Cardiovasc. Diabetol..

[18] ElSayed N.A., Aleppo G., Aroda V.R., Bannuru R.R., Brown F.M., Bruemmer D., Collins B.S., Hilliard M.E., Isaacs D., Johnson E.L. (2023). 2. Classification and Diagnosis of Diabetes: Standards of Care in Diabetes-2023. Diabetes Care.

[19] Ligthart S., van Herpt T.T.W., Leening M.J.G., Kavousi M., Hofman A., Stricker B.H.C., van Hoek M., Sijbrands E.J.G., Franco O.H., Dehghan A. (2016). Lifetime risk of developing impaired glucose metabolism and eventual progression from prediabetes to type 2 diabetes: A prospective cohort study. Lancet Diabetes Endocrinol..

[20] Lewis C., Rafi E., Dobbs B., Barton T., Hatipoglu B., Malin S.K. (2025). Tailoring Exercise Prescription for Effective Diabetes Glucose Management. J. Clin. Endocrinol. Metab..

[21] Korivi M., Mohammed A., Cipryan L., Ye W., Lebaka V.R. (2024). Editorial: Nutritional and physical activity strategies to boost immunity, antioxidant status and health, volume IV. Front. Physiol..

[22] Bin Rakhis S.A., AlDuwayhis N.M., Aleid N., AlBarrak A.N., Aloraini A.A. (2022). Glycemic Control for Type 2 Diabetes Mellitus Patients: A Systematic Review. Cureus.

[23] Sentinelli F., La Cava V., Serpe R., Boi A., Incani M., Manconi E., Solinas A., Cossu E., Lenzi A., Baroni M.G. (2015). Positive effects of Nordic Walking on anthropometric and metabolic variables in women with type 2 diabetes mellitus. Sci. Sports.

[24] Athwale R.M., Shukla M.P. (2024). Effect of supervised nordic walking on glycemic control and maximal aerobic capacity in patients with type 2 diabetes mellitus: A randomized controlled trial. Eur. J. Physiother..

[25] Della Guardia L., Carnevale Pellino V., Filipas L., Bonato M., Gallo G., Lovecchio N., Vandoni M., Codella R. (2023). Nordic Walking Improves Cardiometabolic Parameters, Fitness Performance, and Quality of Life in Older Adults with Type 2 Diabetes. Endocr. Pract..

[26] Sanchez-Lastra M.A., Miller K.J., Martínez-Lemos R.I., Giráldez A., Ayán C. (2020). Nordic Walking for Overweight and Obese People: A Systematic Review and Meta-Analysis. J. Phys. Act. Health.

[27] Liu J., Kim J.H. (2025). The effects of nordic walking on the cardiovascular risk factors in older adults: A systematic review and meta-analysis. Arch. Gerontol. Geriatr..

[28] Bombieri F., Schena F., Pellegrini B., Barone P., Tinazzi M., Erro R. (2017). Walking on four limbs: A systematic review of Nordic Walking in Parkinson disease. Park. Relat. Disord..

[29] Li H., Zhu K., Gan J., Wang Z., Gao Z., Liu L., Guo X., Niu J. (2025). The effects of Nordic walking on cognitive function in older adults: A systematic review and meta-analysis. Front Aging Neurosci..

[30] Khalafi M., Sakhaei M.H., Symonds M.E., Noori Mofrad S.R., Liu Y., Korivi M. (2023). Impact of Exercise in Hypoxia on Inflammatory Cytokines in Adults: A Systematic Review and Meta-analysis. Sports Med. Open.

[31] Page M.J., McKenzie J.E., Bossuyt P.M., Boutron I., Hoffmann T.C., Mulrow C.D., Shamseer L., Tetzlaff J.M., Akl E.A., Brennan S.E. (2021). The Gram et al 2010 statement: An updated guideline for reporting systematic reviews. BMJ.

[32] Bacha F., Hannon T.S., Tosur M., Pike J.M., Butler A., Tommerdahl K.L., Zeitler P.S. (2024). Pathophysiology and Treatment of Prediabetes and Type 2 Diabetes in Youth. Diabetes Care.

[33] Fritz T., Caidahl K., Krook A., Lundström P., Mashili F., Osler M., Szekeres F.L.M., Östenson C.G., Wändell P., Zierath J.R. (2013). Effects of Nordic walking on cardiovascular risk factors in overweight individuals with type 2 diabetes, impaired or normal glucose tolerance. Diabetes/Metab. Res. Rev..

[34] Venojarvi M., Wasenius N., Manderoos S., Heinonen O.J., Hernelahti M., Lindholm H., Surakka J., Lindström J., Aunola S., Atalay M. (2012). Nordic walking decreased circulating chemerin and leptin concentrations in middle-aged men with impaired glucose regulation. Ann. Med..

[35] Kawamoto R., Katoh T., Ninomiya D., Kumagi T., Abe M., Kohara K. (2016). Synergistic association of changes in serum uric acid and triglycerides with changes in insulin resistance after walking exercise in community-dwelling older women. Endocr. Res..

[36] Pippi R., Di Blasio A., Aiello C., Fanelli C., Bullo V., Gobbo S., Cugusi L., Bergamin M. (2020). Effects of a Supervised Nordic Walking Program on Obese Adults with and Without Type 2 Diabetes: The C.U.R.I.A.Mo. Centre Experience. J. Funct. Morphol..

[37] Jabardo-Camprubi G., Puig-Ribera A., Donat-Roca R., Farrés-Godayol P., Nazar-Gonzalez S., Sitjà-Rabert M., Espelt A., Bort-Roig J. (2023). Assessing the Feasibility and Acceptability of a Primary Care Socio-Ecological Approach to Improve Physical Activity Adherence Among People with Type 2 Diabetes: The SENWI Project. Healthcare.

[38] Sterne J.A.C., Savović J., Page M.J., Elbers R.G., Blencowe N.S., Boutron I., Cates C.J., Cheng H.Y., Corbett M.S., Eldridge S.M. (2019). RoB 2: A revised tool for assessing risk of bias in randomised trials. BMJ.

[39] Slim K., Nini E., Forestier D., Kwiatkowski F., Panis Y., Chipponi J. (2003). Methodological index for non-randomized studies (MINORS): Development and validation of a new instrument. ANZ J. Surg..

[40] Higgins J.P.T., Thompson S.G., Deeks J.J., Altman D.G. (2003). Measuring inconsistency in meta-analyses. BMJ.

[41] Deeks J.J., Higgins J.P., Altman D.G., on behalf of the Cochrane Statistical Methods Group (2019). Chapter 10. Analysing Data and Undertaking Meta-Analyses. Cochrane Handbook for Systematic Reviews of Interventions.

[42] Ploydang T., Khovidhunkit W., Tanaka H., Suksom D. (2023). Nordic Walking in Water on Cerebrovascular Reactivity and Cognitive Function in Elderly Patients with Type 2 Diabetes. Med. Sci. Sports Exerc..

[43] Kantorowicz M., Szymura J., Szygula Z., Kusmierczyk J., Maciejczyk M., Wiecek M. (2021). Nordic Walking at Maximal Fat Oxidation Intensity Decreases Circulating Asprosin and Visceral Obesity in Women with Metabolic Disorders. Front. Physiol..

[44] Vehí C., Falces C., Sarlat M.À., Gonzalo A., Andrea R., Sitges M. (2016). Nordic walking for cardiovascular prevention in patients with ischaemic heart disease or metabolic syndrome. Med. Clínica (Engl. Ed.).

[45] Fritz T., Caidahl K., Osler M., Östenson C.G., Zierath J.R., Wändell P. (2011). Effects of Nordic walking on health-related quality of life in overweight individuals with Type 2 diabetes mellitus, impaired or normal glucose tolerance. Diabet. Med..

[46] Rebryna A., Karpiuk I., Obeziuk T., Lyakhova N.Y.A., Rastorguyeva I., Kara I. (2022). Features of Physical Therapy of People with Endocrine System Pathology. Acta Balneol..

[47] Torri A., Volpato E., Merati G., Milani M., Toccafondi A., Formenti D., La Rosa F., Agostini S., Agliardi C., Oreni L. (2024). The VENERE Study: EffectiVenEss of a Rehabilitation Treatment with Nordic Walking in ObEse or OveRweight Diabetic PatiEnts with Cardiovascular Disease. CJC Open.

[48] Fiodorenko-Dumas Z., Dumas I., Mastej K., Adamiec R. (2017). Physical activity—Related changes in ADMA and vWF levels in patients with type 2 diabetes—A preliminary study. Adv. Clin. Exp. Med..

[49] Özdamar M., Erkek Ö., TÜMkaya S., ÖZdamar H., ÖZdamar A., Pakyurek H., Tunç Ata M., Senol H., Kilic-Toprak E., Bor-Kucukatay M. (2022). Examination of the Effectiveness of 12-Week Nordic Walking Exercise in Prediabetic Individuals. Pamukkale Med. J..

[50] Neumayr G., Engler C., Lunger L., Lechleitner P. (2020). Effects of a One-Week Vacation with Various Activity Programs on Metabolism and Adipokines. Int. J. Sports Med..

[51] Du X., Zhang C., Zhang X., Qi Z., Cheng S., Le S. (2021). The Impact of Nordic Walking on Bone Properties in Postmenopausal Women with Pre-Diabetes and Non-Alcohol Fatty Liver Disease. Int. J. Environ. Res. Public Health.

[52] Venojarvi M., Korkmaz A., Wasenius N., Manderoos S., Heinonen O.J., Lindholm H., Aunola S., Eriksson J.G., Atalay M. (2013). 12 Weeks’ aerobic and resistance training without dietary intervention did not influence oxidative stress but aerobic training decreased atherogenic index in middle-aged men with impaired glucose regulation. Food Chem. Toxicol..

[53] Ring M., Eriksson M.J., Fritz T., Nyberg G., Östenson C.G., Krook A., Zierath J.R., Caidahl K. (2015). Influence of physical activity and gender on arterial function in type 2 diabetes, normal and impaired glucose tolerance. Diabetes Vasc. Dis. Res..

[54] Gidlund E.-K., von Walden F., Venojärvi M., Risérus U., Heinonen O.J., Norrbom J., Sundberg C.J. (2016). Humanin skeletal muscle protein levels increase after resistance training in men with impaired glucose metabolism. Physiol. Rep..

[55] Canoy D., Nazarzadeh M., Copland E., Bidel Z., Rao S., Li Y., Rahimi K. (2022). How Much Lowering of Blood Pressure Is Required to Prevent Cardiovascular Disease in Patients with and Without Previous Cardiovascular Disease?. Curr. Cardiol. Rep..

[56] Lewington S., Clarke R., Qizilbash N., Peto R., Collins R. (2002). Age-specific relevance of usual blood pressure to vascular mortality: A meta-analysis of individual data for one million adults in 61 prospective studies. Lancet.

[57] Higgins J.P.T., Li T. (2022). Exploring Heterogeneity. Systematic Reviews in Health Research.

[58] Lockett A. (2025). Systematic review and meta-analysis in clinical trials. Medicine.

[59] Sun Y., Liu Y., Ye W., Lebaka V.R., Chenji V., Li W., Korivi M. (2025). Efficiency of time-restricted eating and energy restriction on anthropometrics and body composition in adults: A systematic review and meta-analysis of randomized controlled trials. Int. J. Behav. Nutr. Phys. Act..

[60] Khurana A., Taksande A., Meshram R.J. (2024). Beyond Boundaries: A Comprehensive Review of Anthropometric Indices in Urban and Rural India. Cureus.

[61] Davenport M.H., Ruchat S.-M., Jaramillo Garcia A., Ali M.U., Forte M., Beamish N., Fleming K., Adamo K.B., Brunet-Pagé É., Chari R. (2025). 2025 Canadian guideline for physical activity, sedentary behaviour and sleep throughout the first year post partum. Br. J. Sports Med..

[62] Jain A., Kristensen P.N., Wasehuus R.S., Kruse C., Nørtoft E. (2026). Clinical implications of intentional weight loss in people living with type 2 diabetes: A real-world database study. Diabetes Obes. Metab..

[63] Gray N., Picone G., Sloan F., Yashkin A. (2015). Relation Between BMI and Diabetes Mellitus and Its Complications Among US Older Adults. South. Med. J..

[64] Sharafi M., Mohsenpour M.A., Afrashteh S., Eftekhari M.H., Dehghan A., Farhadi A., Jafarnezhad A., Zakeri A., Looha M.A. (2024). Factors affecting the survival of prediabetic patients: Comparison of Cox proportional hazards model and random survival forest method. BMC Med. Inform. Decis. Mak..

[65] Schiffer T., Knicker A., Hoffman U., Harwig B., Hollmann W., Strüder H.K. (2006). Physiological responses to nordic walking, walking and jogging. Eur. J. Appl. Physiol..

[66] Sarwar N., Gao P., Seshasai S.R., Gobin R., Kaptoge S., Di Angelantonio E., Ingelsson E., Lawlor D.A., Selvin E., Stampfer M. (2010). Diabetes mellitus, fasting blood glucose concentration, and risk of vascular disease: A collaborative meta-analysis of 102 prospective studies. Lancet.

[67] Kawamoto R., Katoh T., Kohara K., Miki T. (2015). Determinants of change in insulin resistance response to Nordic walking in community-dwelling elderly women. J. Clin. Gerontol. Geriatr..

[68] Makris K., Spanou L. (2011). Is there a relationship between mean blood glucose and glycated hemoglobin?. Diabetes Sci. Technol..

[69] Kilpatrick E.S. (2008). Haemoglobin A1c in the diagnosis and monitoring of diabetes mellitus. J. Clin. Pathol..

[70] Nathan D.M. (1993). Long-term complications of diabetes mellitus. N. Engl. J. Med..

[71] Yang T., Qi F., Guo F., Shao M., Song Y., Ren G., Linlin Z., Qin G., Zhao Y. (2024). An update on chronic complications of diabetes mellitus: From molecular mechanisms to therapeutic strategies with a focus on metabolic memory. Mol. Med..

[72] Cowart K., Carris N.W. (2025). Current treatment guidelines and glycated haemoglobin goals for type 2 diabetes: Which patients are most likely to benefit from fixed-ratio basal insulin glucagon-like peptide-1 receptor agonist combinations?. Diabetes Obes. Metab..

[73] Chudyk A., Petrella R.J. (2011). Effects of exercise on cardiovascular risk factors in type 2 diabetes: A meta-analysis. Diabetes Care.

[74] Snowling N.J., Hopkins W.G. (2006). Effects of different modes of exercise training on glucose control and risk factors for complications in type 2 diabetic patients: A meta-analysis. Diabetes Care.

[75] Manzoli L., Qiu S., Cai X., Schumann U., Velders M., Sun Z., Steinacker J.M. (2014). Impact of Walking on Glycemic Control and Other Cardiovascular Risk Factors in Type 2 Diabetes: A Meta-Analysis. PLoS ONE.

[76] Liu Y., Ye W., Chen Q., Zhang Y., Kuo C.H., Korivi M. (2019). Resistance Exercise Intensity is Correlated with Attenuation of HbA1c and Insulin in Patients with Type 2 Diabetes: A Systematic Review and Meta-Analysis. Int. J. Environ. Res. Public Health.

[77] Khalafi M., Mojtahedi S., Ostovar A., Rosenkranz S.K., Korivi M. (2022). High-intensity interval exercise versus moderate-intensity continuous exercise on postprandial glucose and insulin responses: A systematic review and meta-analysis. Obes. Rev..

[78] Richter E.A., Hargreaves M. (2013). Exercise, GLUT4, and skeletal muscle glucose uptake. Physiol. Rev..

[79] Bird S.R., Hawley J.A. (2017). Update on the effects of physical activity on insulin sensitivity in humans. BMJ Open Sport Exerc. Med..

[80] Stanford K.I., Goodyear L.J. (2014). Exercise and type 2 diabetes: Molecular mechanisms regulating glucose uptake in skeletal muscle. Adv. Physiol. Educ..

[81] Yao X., Xia J., Deodat M., Wang P., Chang Y., Luo Y., Ji L. (2025). GRADE notes 6: Three strategies to determine the clinically important thresholds for outcomes in evidence-based guideline development. J. Clin. Epidemiol..

[82] Dankers M., Nelissen-Vrancken M., Hart B.H., Lambooij A.C., van Dijk L., Mantel-Teeuwisse A.K. (2021). Alignment between outcomes and minimal clinically important differences in the Dutch type 2 diabetes mellitus guideline and healthcare professionals’ preferences. Pharmacol. Res. Perspect..

[83] Chiavaroli L., Lee D., Ahmed A., Cheung A., Khan T.A., Blanco S., Mejia, Mirrahimi A., Jenkins D.J.A., Livesey G. (2021). Effect of low glycaemic index or load dietary patterns on glycaemic control and cardiometabolic risk factors in diabetes: Systematic review and meta-analysis of randomised controlled trials. BMJ.

[84] Stratton I.M., Adler A.I., Neil H.A.W., Matthews D.R., Manley S.E., Cull C.A., Hadden D., Turner R.C., Holman R.R. (2000). Association of glycaemia with macrovascular and microvascular complications of type 2 diabetes (UKPDS 35): Prospective observational study. BMJ.

[85] Goldenberg J.Z., Day A., Brinkworth G.D., Sato J., Yamada S., Jönsson T., Beardsley J., Johnson J.A., Thabane L., Johnston B.C. (2021). Efficacy and safety of low and very low carbohydrate diets for type 2 diabetes remission: Systematic review and meta-analysis of published and unpublished randomized trial data. BMJ.

[86] Prusik K., Kortas J., Prusik K., Mieszkowski J., Jaworska J., Skrobot W., Lipinski M., Ziemann E., Antosiewicz J. (2018). Nordic Walking Training Causes a Decrease in Blood Cholesterol in Elderly Women Supplemented with Vitamin D. Front. Endocrinol..

[87] Czerwińska-Ledwig O., Kryst J., Ziemann E., Borkowska A., Reczkowicz J., Dzidek A., Rydzik Ł., Pałka T., Żychowska M., Kupczak W. (2024). The Beneficial Effects of Nordic Walking Training Combined with Time-Restricted Eating 14/24 in Women with Abnormal Body Composition Depend on the Application Period. Nutrients.

[88] Bullo V., Gobbo S., Vendramin B., Duregon F., Cugusi L., Di Blasio A., Bocalini D.S., Zaccaria M., Bergamin M., Ermolao A. (2017). Nordic Walking Can Be Incorporated in the Exercise Prescription to Increase Aerobic Capacity, Strength, and Quality of Life for Elderly: A Systematic Review and Meta-Analysis. Rejuvenation Res..

[89] Wang J., Tan S., Cao L. (2015). Exercise training at the maximal fat oxidation intensity improved health-related physical fitness in overweight middle-aged women. J. Exerc. Sci. Fit..

[90] Cebula A., Tyka A.K., Tyka A., Pałka T., Pilch W., Luty L., Mucha D. (2020). Physiological response and cardiorespiratory adaptation after a 6-week Nordic Walking training targeted at lipid oxidation in a group of post-menopausal women. PLoS ONE.

[91] Huta-Osiecka A., Wochna K., Stemplewski R., Marciniak K., Podgórski T., Kasprzak Z., Leszczyński P., Nowak A. (2022). Influence of Nordic walking with poles with an integrated resistance shock absorber on carbohydrate and lipid metabolic indices and white blood cell subpopulations in postmenopausal women. PeerJ.

[92] Kwon H.J., Lee H.J. (2017). Effect of vigorous physical activity on blood lipid and glucose. J. Exerc. Rehabil..

[93] Mann S., Beedie C., Jimenez A. (2014). Differential effects of aerobic exercise, resistance training and combined exercise modalities on cholesterol and the lipid profile: Review, synthesis and recommendations. Sports Med..

[94] Kraus W.E., Houmard J.A., Duscha B.D., Knetzger K.J., Wharton M.B., McCartney J.S., Bales C.W., Henes S., Samsa G.P., Otvos J.D. (2002). Effects of the amount and intensity of exercise on plasma lipoproteins. N. Engl. J. Med..

[95] Nybo L., Sundstrup E., Jakobsen M.D., Mohr M., Hornstrup T., Simonsen L., Bülow J., Randers M.B., Nielsen J.J., Aagaard P. (2010). High-intensity training versus traditional exercise interventions for promoting health. Med. Sci. Sports Exerc..

[96] Fuchs F.D., Whelton P.K. (2020). High Blood Pressure and Cardiovascular Disease. Hypertension.

[97] Piskorz D. (2020). Hypertension and metabolic disorders, a glance from different phenotypes. Am. J. Prev. Cardiol..

[98] Glandt M., Bloomgarden Z.T. (2011). Hypertension in diabetes: Treatment considerations. J. Clin. Hypertens. (Greenwich).

[99] Greeff D. (2006). An approach to preventing and treating hypertension through lifestyle modification: Clinical. SA Pharm. J..

[100] Trillaud E., Klemmer P., Malin S.K., Erdbrügger U. (2023). Tracking Biomarker Responses to Exercise in Hypertension. Curr. Hypertens. Rep..

[101] Sabbahi A., Arena R., Elokda A., Phillips S.A. (2016). Exercise and Hypertension: Uncovering the Mechanisms of Vascular Control. Prog. Cardiovasc. Dis..

[102] Wang Y., Parpia S., Couban R., Wang Q., Armijo-Olivo S., Bassler D., Briel M., Brignardello-Petersen R., Gluud L.L., Keitz S.A. (2024). Compelling evidence from meta-epidemiological studies demonstrates overestimation of effects in randomized trials that fail to optimize randomization and blind patients and outcome assessors. J. Clin. Epidemiol..

[103] Bialy L., Vandermeer B., Lacaze-Masmonteil T., Dryden D.M., Hartling L. (2014). A meta-epidemiological study to examine the association between bias and treatment effects in neonatal trials. Evid.-Based Child. Health.

