# Supplementary material for: Changes in Cardiometabolic Risk Factors Following Nordic Walking in Adults with Prediabetes or Diabetes: A Systematic Review and Meta-Analysis of Pre–Post Interventions
Source: Life (Basel). 2026 Jul 13;16(7):1159. doi: 10.3390/life16071159 (PMC13413405; doi:10.3390/life16071159)
Supplement: Supplementary file 1 [file life-16-01159-s001.zip › life-4372719-supplementary.pdf]

Changes in Cardiometabolic Risk Factors following Nordic Walking in Adults with Prediabetes or Diabetes: A Systematic Review and Meta-Analysis of Pre-Post Interventions

Ankang Wu [1], Sichao Chen [1], Yubo Liu [1], Yong Zhang [2], Mallikarjuna Korivi [1,*] and Weibing Ye [1,*]

Supplementary Materials

Supplementary Table S1. Detailed search strategy for each database.

Database	Query	Results
**PubMed**	(((("Nordic Walking"[Mesh]) OR (Walking, Nordic)) OR (Pole Walking)) OR (Walking, Pole)) AND (("Diabetes Mellitus, Type 2"[Mesh]) OR (Diabetes Mellitus)) OR (Diabetes Mellitus, Noninsulin Dependent)) OR (Diabetes Mellitus, Adult Onset)) OR (Adult Onset Diabetes Mellitus)) OR (Diabetes Mellitus, Ketosis-Resistant)) OR (Diabetes Mellitus, Ketosis Resistant)) OR (Ketosis-Resistant Diabetes Mellitus)) OR (Diabetes Mellitus, Non-Insulin-Dependent)) OR (Non-Insulin-Dependent Diabetes Mellitus)) OR (Diabetes Mellitus, Type II)) OR (NIDDM)) OR (Diabetes Mellitus, Maturity-Onset)) OR (Diabetes Mellitus, Maturity Onset)) OR (Maturity-Onset Diabetes Mellitus)) OR (Maturity Onset Diabetes Mellitus)) OR (MODY)) OR (Diabetes Mellitus, Slow-Onset)) OR (Diabetes Mellitus, Slow Onset)) OR (Slow-Onset Diabetes Mellitus)) OR (Type 2 Diabetes Mellitus)) OR (Noninsulin-Dependent Diabetes Mellitus)) OR (Noninsulin Dependent Diabetes Mellitus)) OR (Maturity-Onset Diabetes)) OR (Diabetes, Maturity-Onset)) OR (Maturity Onset Diabetes)) OR (T2DM)) OR (Type 2 Diabetes)) OR (Diabetes, Type 2)) OR (Diabetes Mellitus, Noninsulin-Dependent)) OR (Insulin Resistance)) OR (Resistance, Insulin)) OR (Insulin Sensitivity)) OR (Sensitivity, Insulin)) OR (Glucose Intolerance)) OR (Glucose Intolerances)) OR (Glucose Intolerances)) OR (Intolerances, Glucose)) OR (Impaired Glucose Tolerance)) OR (Glucose Tolerance, Impaired)) OR (Glucose Tolerances, Impaired)) OR (Impaired Glucose Tolerances) OR (Tolerance, Impaired Glucose)) OR (Tolerances, Impaired Glucose)) OR (homeostasis model assessment of insulin resistance)) OR (HOMA-IR))	34
**Web of Science**	(TS=(Nordic Walking) OR AB=(Walking, Nordic OR Pole Walking OR Walking, Pole)) AND (TS=(Diabetes Mellitus, Type 2) OR AB=(Diabetes Mellitus, Stable OR Stable Diabetes Mellitus OR Diabetes Mellitus, Noninsulin Dependent OR Diabetes Mellitus, Adult-Onset OR Adult-Onset Diabetes Mellitus OR Diabetes Mellitus, Adult Onset OR Diabetes Mellitus, Ketosis-Resistant OR Diabetes Mellitus, Ketosis Resistant OR Ketosis-Resistant Diabetes Mellitus OR Diabetes Mellitus, Non Insulin Dependent OR Diabetes Mellitus, Non-Insulin-Dependent OR Non-Insulin-Dependent Diabetes Mellitus OR Diabetes Mellitus, Type II OR NIDDM OR Diabetes Mellitus, Maturity-Onset OR Diabetes Mellitus,	75

	Maturity Onset OR Maturity-Onset Diabetes Mellitus OR Maturity Onset Diabetes Mellitus OR MODY OR Diabetes Mellitus, Slow-Onset OR Diabetes Mellitus, Slow Onset OR Slow-Onset Diabetes Mellitus OR Type 2 Diabetes Mellitus OR Noninsulin-Dependent Diabetes Mellitus OR Noninsulin Dependent Diabetes Mellitus OR Maturity-Onset Diabetes OR Diabetes, Maturity-Onset OR Maturity Onset Diabetes OR Type 2 Diabetes OR Diabetes, Type 2 OR T2DM OR Diabetes Mellitus, Noninsulin-Dependent OR Insulin Resistance OR Impaired Glucose Tolerance OR homeostasis model assessment of insulin resistance OR HOMA-IR))	
**The Cochrane**	#15 MeSH descriptor: [Nordic Walking] explode all trees<br>#16 ("Nordic Walking"):ti,ab,kw OR ("Walking, Nordic"):ti,ab,kw OR ("Pole Walking"):ti,ab,kw OR ("Walking, Pole"):ti,ab,kw<br>#17 MeSH descriptor: [Diabetes Mellitus, Type 2] explode all trees<br>#18 (Diabetes Mellitus, Type 2):ti,ab,kw OR (Diabetes Mellitus, Stable):ti,ab,kw OR (Stable Diabetes Mellitus):ti,ab,kw OR (Diabetes Mellitus, Noninsulin Dependent):ti,ab,kw OR (Diabetes Mellitus, Adult-Onset):ti,ab,kw OR (Adult-Onset Diabetes Mellitus):ti,ab,kw OR (Diabetes Mellitus, Adult Onset):ti,ab,kw OR (Diabetes Mellitus, Ketosis-Resistant):ti,ab,kw OR (Diabetes Mellitus, Ketosis Resistant):ti,ab,kw OR (Ketosis-Resistant Diabetes Mellitus):ti,ab,kw OR (Diabetes Mellitus, Non Insulin Dependent):ti,ab,kw OR (Diabetes Mellitus, Non-Insulin-Dependent):ti,ab,kw OR (Non-Insulin-Dependent Diabetes Mellitus):ti,ab,kw OR (Diabetes Mellitus, Type II):ti,ab,kw OR (NIDDM):ti,ab,kw OR (Diabetes Mellitus, Maturity-Onset):ti,ab,kw OR (Diabetes Mellitus, Maturity Onset):ti,ab,kw OR (Maturity-Onset Diabetes Mellitus):ti,ab,kw OR (Maturity Onset Diabetes Mellitus):ti,ab,kw OR (MODY):ti,ab,kw OR (Diabetes Mellitus, Slow-Onset):ti,ab,kw OR (Diabetes Mellitus, Slow Onset):ti,ab,kw OR (Slow-Onset Diabetes Mellitus):ti,ab,kw OR (Type 2 Diabetes Mellitus):ti,ab,kw OR (Noninsulin-Dependent Diabetes Mellitus):ti,ab,kw OR (Noninsulin Dependent Diabetes Mellitus):ti,ab,kw OR (Maturity-Onset Diabetes):ti,ab,kw OR (Diabetes, Maturity-Onset):ti,ab,kw OR (Maturity Onset Diabetes):ti,ab,kw OR (Type 2 Diabetes):ti,ab,kw OR (Diabetes, Type 2):ti,ab,kw OR (T2DM):ti,ab,kw OR (Diabetes Mellitus, Noninsulin-Dependent):ti,ab,kw OR (Insulin Resistance):ti,ab,kw OR (Impaired Glucose Tolerance):ti,ab,kw OR (homeostasis model assessment of insulin resistance):ti,ab,kw OR (HOMA-IR):ti,ab,kw<br>#15 OR #16<br>#17 OR #18<br>#19 AND #20	36
**Scopus**	((TITLE-ABS-KEY ("Walking, Nordic") OR TITLE-ABS-KEY ("Pole Walking") OR TITLE-ABS-KEY ("Walking, Pole") OR TITLE-ABS-KEY	45

	("Nordic Walking"))) AND ((TITLE-ABS-KEY ("Diabetes Mellitus, Stable") OR TITLE-ABS-KEY ("Stable Diabetes Mellitus") OR TITLE-ABS-KEY ("Diabetes Mellitus, Noninsulin Dependent") OR TITLE-ABS-KEY ("Diabetes Mellitus, Adult-Onset") OR TITLE-ABS-KEY ("Adult-Onset Diabetes Mellitus") OR TITLE-ABS-KEY ("Diabetes Mellitus, Adult Onset") OR TITLE-ABS-KEY ("Diabetes Mellitus, Ketosis-Resistant") OR TITLE-ABS-KEY ("Diabetes Mellitus, Ketosis Resistant") OR TITLE-ABS-KEY ("Ketosis-Resistant Diabetes Mellitus") OR TITLE-ABS-KEY ("Diabetes Mellitus, Non Insulin Dependent") OR TITLE-ABS-KEY ("Diabetes Mellitus, Non-Insulin-Dependent") OR TITLE-ABS-KEY ("Non-Insulin-Dependent Diabetes Mellitus") OR TITLE-ABS-KEY ("Diabetes Mellitus, Type II") OR TITLE-ABS-KEY (NIDDM) OR TITLE-ABS-KEY ("Diabetes Mellitus, Maturity-Onset") OR TITLE-ABS-KEY ("Diabetes Mellitus, Maturity Onset") OR TITLE-ABS-KEY ("Maturity-Onset Diabetes Mellitus") OR TITLE-ABS-KEY ("Maturity Onset Diabetes Mellitus") OR TITLE-ABS-KEY (MODY) OR TITLE-ABS-KEY ("Diabetes Mellitus, Slow-Onset") OR TITLE-ABS-KEY ("Diabetes Mellitus, Slow Onset") OR TITLE-ABS-KEY ("Slow-Onset Diabetes Mellitus") OR TITLE-ABS-KEY ("Type 2 Diabetes Mellitus") OR TITLE-ABS-KEY ("Noninsulin-Dependent Diabetes Mellitus") OR TITLE-ABS-KEY ("Noninsulin Dependent Diabetes Mellitus") OR TITLE-ABS-KEY ("Maturity-Onset Diabetes") OR TITLE-ABS-KEY ("Diabetes, Maturity-Onset") OR TITLE-ABS-KEY ("Maturity Onset Diabetes") OR TITLE-ABS-KEY ("Type 2 Diabetes") OR TITLE-ABS-KEY ("Diabetes, Type 2") OR TITLE-ABS-KEY (T2DM) OR TITLE-ABS-KEY ("Diabetes Mellitus, Noninsulin-Dependent") OR TITLE-ABS-KEY ("Insulin Resistance") OR TITLE-ABS-KEY ("Impaired Glucose Tolerance") OR TITLE-ABS-KEY ("homeostasis model assessment of insulin resistance") OR TITLE-ABS-KEY (HOMA-IR)))	
**Embase**	#1 'nordic walking'/exp OR 'nordic walking' OR 'walking, nordic' OR 'pole walking'/exp OR 'pole walking' OR 'walking, pole'<br>#2 'diabetes mellitus, type' OR 'diabetes mellitus, stable' OR 'stable diabetes mellitus' OR 'diabetes mellitus, noninsulin dependent' OR 'diabetes mellitus, adult-onset' OR 'adult-onset diabetes mellitus'/exp OR 'adult-onset diabetes mellitus' OR 'diabetes mellitus, adult onset' OR 'diabetes mellitus, ketosis-resistant' OR 'diabetes mellitus, ketosis resistant' OR 'ketosis-resistant diabetes mellitus'/exp OR 'ketosis-resistant diabetes mellitus' OR 'diabetes mellitus, non insulin dependent'/exp OR 'diabetes mellitus, non insulin dependent' OR 'diabetes mellitus, non-insulin-dependent'/exp OR 'diabetes mellitus, non-insulin-dependent' OR 'non-insulin-dependent diabetes mellitus'/exp OR 'non-insulin-dependent diabetes mellitus' OR 'diabetes mellitus, type ii'/exp OR 'diabetes mellitus, type ii' OR 'niddm'/exp OR	60

	'niddm' OR 'diabetes mellitus, maturity-onset'/exp OR 'diabetes mellitus, maturity-onset' OR 'diabetes mellitus, maturity onset'/exp OR 'diabetes mellitus, maturity onset' OR 'maturity-onset diabetes mellitus'/exp OR 'maturity-onset diabetes mellitus' OR 'maturity onset diabetes mellitus'/exp OR 'maturity onset diabetes mellitus' OR 'mody' OR 'diabetes mellitus, slow-onset' OR 'diabetes mellitus, slow onset' OR 'slow-onset diabetes mellitus' OR 'type 2 diabetes mellitus'/exp OR 'type 2 diabetes mellitus' OR 'noninsulin-dependent diabetes mellitus'/exp OR 'noninsulin-dependent diabetes mellitus' OR 'noninsulin dependent diabetes mellitus'/exp OR 'noninsulin dependent diabetes mellitus' OR 'maturity-onset diabetes'/exp OR 'maturity-onset diabetes' OR 'diabetes, maturity-onset' OR 'maturity onset diabetes'/exp OR 'maturity onset diabetes' OR 'type 2 diabetes'/exp OR 'type 2 diabetes' OR 'diabetes, type 2'/exp OR 'diabetes, type 2' OR 'diabetes mellitus, noninsulin-dependent' OR 'Impaired Glucose Tolerance' OR 'Insulin Resistance' OR 'homeostasis model assessment of insulin resistance' OR 'HOMA-IR'<br>#1 AND #2	

Supplementary Table S2. Comparator conditions of the included studies

Study	Study design	Intervention trial	Control trial
Gram et al., 2010 [11]	RCT	Nordic Walking	Structured exercise program
Fritz et al., 2013 [33]	RCT	Nordic Walking	Habitual physical activity
Venojarvi et al., 2013 [34]	RCT	Nordic Walking	No supervised exercise
Sentinelli et al., 2015 [23]	RCT	Nordic Walking	Physical activity counseling
Kawamoto et al., 2016 [35]	Non-RCT	Nordic Walking	None
Pippi et al., 2020 [36]	Non-RCT	Nordic Walking	Gym-based exercise
Jabardo-Camprubi et al., 2023 [37]	RCT	Nordic Walking	Physical activity counseling
Della Guardia et al., 2023 [25]	Non-RCT	Nordic Walking	None
Athwale and Shukla 2024 [24]	RCT	Nordic Walking	Physical activity counseling
